# Editorial: Myopia in childhood and adolescence

**DOI:** 10.3389/fopht.2025.1755753

**Published:** 2026-01-20

**Authors:** Claudia Carolina Cruz-Gálvez, Juan Carlos Ordaz-Favila, Vanessa Bosch-Canto

**Affiliations:** 1 Physiology Department, Centro Universitario de Ciencias de la Salud, Universidad de Guadalajara, Guadalajara, Mexico; 2 Pediatric Ophthalmology, Instituto Nacional de Pediatría, Universidad Autónoma de México, México City, Mexico

**Keywords:** adolescence, childhood, myopia, myopia control, myopia correction, myopia epidemic, myopia management, public health

Myopia has emerged as a significant public health concern over the past few decades. Once considered a condition primarily affecting school-aged children, its prevalence has surged alarmingly across all age groups and geographical regions. And its long-term complications have become a huge concern. It is expected that in the second half of this century, the visual impairment due to myopia will increase significantly. This editorial has 2 main guidelines: myopia management and myopia control, aspects that are addressed in this Research Topic about *Myopia in Childhood and Adolescence*, which is made up of 16 articles with the participation of 94 authors.

According to the International Myopia Institute (IMI) White Papers IV 2025, Myopia management is a comprehensive eye and vision care approach to myopia and pre-myopia that includes prevention, myopia risk assessment, early detection through screening, appropriate correction, lifestyle recommendations, interventions to reduce myopia progression and axial elongation, monitoring of refraction and axial length, and management of emerging myopia-related complications. Myopia control constitutes one essential component within the broader scope of myopia management and should include monitoring of treatment efficacy. Myopia Correction refers to devices and interventions that correct the optical focusing errors of myopia to optimize best corrected distance visual acuity, without providing any intended benefits in relation to slowing myopia progression or axial elongation.

Recent epidemiological studies reveal a staggering increase in myopia worldwide. In this Research Topic, Hönekopp et al. investigate the prevalence of myopia and uncorrected myopia among primary and secondary school students in Germany, and analyze potential sociodemographic predictors for refractive status. Hu et al. determined the prevalence of myopia among children in Shihezi City, considered the effects of gender and urban–rural distribution on myopia, and explored the influencing factors of myopia to fill the gaps in existing knowledge about myopia in China. Wang et al. report the prevalence of myopia among children and adolescents in Jianyang City, China.

In East Asian countries, the prevalence of myopia among young adults has reached upwards of 80%, with severe cases manifesting in forms that may induce debilitating complications such as retinal detachment, myopic macular degeneration, and glaucoma.

The World Health Organization (WHO) has classified myopia and its associated complications as concerning public health threats, particularly as they can lead to significant visual impairment in young adults and have financial and health implications.

The surge in myopia prevalence can be attributed to a combination of genetic, environmental, and lifestyle factors:

- Genetic Predisposition: Family history has long been recognized as a risk factor for myopia. Children with myopic parents are at a significantly higher risk of developing the condition themselves.
- Environmental Influences: A pivotal shift towards more indoor activities has been observed, particularly in urban settings. Prolonged screen time associated with academic engagement and leisure activities limits exposure to natural sunlight, which plays a protective role in ocular health.
- Education and Visual Demand: Increasing educational pressures and the demand for near-vision tasks have been correlated with higher myopia rates. This intensification of visual demands, particularly during formative years, could be expediting the development of myopia in the younger population.

Based on the above, Qin et al. elucidate, through a survey, the effects of genetic, growth, and development, and environmental factors on the ocular biometric parameters of preschool children in Beijing, China. Zhong et al. investigate the associations between adherence to the Canadian 24-Hour Movement Guidelines for Children and Youth—covering physical activity (PA), screen time (ST), and sleep duration (SD)—and myopia and myopic anisometropia, among children and adolescents in Shenzhen, China. Iyer et al. assess the separate and combined effects of preterm birth and screen time on spectacle wear among 5-year-olds and adolescents. Early preterm-born (EP< 32 weeks) and Moderately-late Preterm-born (MLP 32–36 weeks) children have a significantly increased risk of spectacle wear at age 5, and at that age, the risk of spectacle wear decreased by 7% for each additional week of gestational age.

Ocular biometric parameters are essential in myopia management and myopia control. Cao et al. describe the progression of myopia and associated factors of axial length (AL) growth among children. They point out that AL serves as a more sensitive indicator for monitoring myopia progression, the anterior chamber depth (ACD) has positively correlated with AL growth, and deeper ACD may contribute to longer AL growth. Gao et al. investigated changes in ocular parameters and developed machine learning-based models to predict myopia onset and progression in school-aged pre-myopes.

Aberrometry has significant implications in myopia management. Mohaghegh et al. investigate associations between refractive error components and higher-order aberrations (HOAs) in adult myopic subjects. They observe that total RMS wave-front error increases with increasing myopia and astigmatism, and that the increasing myopia power does not show a systematic correlation with HOAs components.

The implications of rising myopia prevalence extend beyond mere visual impairment. Myopia-related complications present a looming threat to global health systems, resulting in increased healthcare expenditures, loss of productivity, and diminished quality of life for affected individuals. Furthermore, as populations age, the burden of myopia and its sequelae is projected to exacerbate, necessitating urgent attention from policymakers and public health officials. It is imperative to understand this epidemic and start doing something to control it.

Dan et al. compared the pre- and postoperative best-corrected visual acuity (BCVA) and maximum foveal thickness (MaxFT) of patients with foveal detachment (MFFD) who underwent vitrectomy with silicone oil or perfluoropropane (C3F8) tamponade.

Awareness and education are fundamental in combating the myopia epidemic. Timing is decisive. We have a big opportunity in children to stop the natural progression of high myopia. Instituting comprehensive public health campaigns that advocate for mandatory and routine eye examinations and raise awareness about the importance of outdoor activity is crucial. Schools can play a pivotal role in implementing changes, such as incorporating outdoor recess and limiting screen time.

Additionally, advancements in optical interventions, such as myopia management spectacles, multifocal soft contact lenses, overnight orthokeratology, pharmacologic treatments like atropine, and light-based therapies show promise in controlling myopia progression in children. Research into the genetic underpinnings of myopia may yield new insights that could inform preventative strategies.

Wang et al. analyze the factors influencing the effectiveness of orthokeratology in controlling myopia in children. The findings suggest that baseline patient age and the severity of initial myopia should be considered when predicting treatment outcomes. Zhang et al. developed and validated a predictive model utilizing clinical and ocular measurements to predict the effect of overnight orthokeratology on myopia control through machine learning. Wang et al. evaluate the subfoveal choroidal thickness (SFChT) and choriocapillaris (CC) perfusion in myopic children following orthokeratology usage. Orthokeratology notably increases SFChT and improves CC perfusion. Chen et al. evaluate the efficacy and tolerability of different concentrations of atropine in slowing the myopia progression in adolescents and children. Fan et al. through a meta-analysis, demonstrate that Repeated Low-level Red Light (RLRL) treatment has significant clinical effects on pre-myopic and myopic Chinese children, including increasing subfoveal choroidal thickness, delaying axial length elongation, and reducing spherical equivalent refraction progression. Weiwei et al. estimated the effectiveness of Far-infrared (FIR) therapy in myopia control and explored its underlying mechanisms in Guinea pigs, reporting myopia control effectively.

The rising tide of myopia presents a pressing challenge that requires a multifaceted approach. As a global community, we must prioritize research, education, patient empowerment, bioethics in professionals related to visual health, and policy changes that address both the modifiable risk factors and the underlying causes of myopia. By doing so, we can work towards mitigating this public health crisis that is coming in the next years and improving visual health outcomes for future generations. Through collaboration among governments, national health systems, ophthalmologists, optometrists, researchers, practitioners, educators, and families, we can equip ourselves to tackle the complexities of myopia and safeguard the vision of millions worldwide.

